# A Quality Improvement Project to Standardize Surfactant Delivery in the Era of Noninvasive Ventilation

**DOI:** 10.1097/pq9.0000000000000311

**Published:** 2020-06-26

**Authors:** Jeong Eun Kim, Mariana Brewer, Regina Spinazzola, Elfriede Wallace, Joanne Casatelli, Joanna Beachy, Barry Weinberger, Shahana Perveen

**Affiliations:** From the * Cohen Children’s Medical Center, New Hyde Park, N.Y.; † North Shore University Hospital, Manhasset, N.Y.; ‡ Zucker School of Medicine at Hofstra/Northwell, Hempstead, N.Y.

## Abstract

**Introduction:**

Continuous positive airway pressure (CPAP) and surfactant both improve outcomes for premature infants with respiratory distress syndrome. However, prolonged trials of CPAP, as well as observation periods after intubation, may delay the administration of surfactant. Late surfactant treatment likely increases the incidence of bronchopulmonary dysplasia, which leads to significant morbidity and healthcare utilization.

**Methods:**

We aimed to decrease time from meeting standard criteria (start of a continuous run of F_i_O_2_ > 40% or P_a_CO_2_ > 65 for >90 min) to intubation, and from intubation to surfactant administration, for infants <1,500 g or younger than 32 weeks gestation. Retrospective data collection from the electronic medical record assessed those process measures as the primary endpoints. Balancing measures were the adverse outcomes of asymmetric lung disease, the inappropriate position of the endotracheal tube, or pneumothorax on the first x-ray (within 24 h) after surfactant.

**Results:**

Mean time to intubation for infants 28–32 weeks gestation decreased from 321 to 81 minutes in response to a literature review for physicians and free-text orders for notification. Time to intubation for infants younger than 28 weeks gestation did not change. Administration of surfactant within 1 hour of intubation improved from 78% to 100% after a program for trainees and coordination with radiology. There were no adverse occurrences.

**Conclusions:**

Educational interventions and targeted process change can successfully implement standard criteria for intubation and surfactant administration for premature infants. Determination of an acceptable range of evidence-based practice is essential for the engagement of medical staff. Timely intubation and surfactant may decrease bronchopulmonary dysplasia.

## INTRODUCTION

Bronchopulmonary dysplasia (BPD) is one of the most common complications experienced by very-low-birth-weight (VLBW) infants (younger than 32 weeks gestation and 1,500 g birth weight) and is associated with high morbidity and mortality. The risk of developing BPD is related to mechanical, oxidative, and inflammatory injury to the immature lung, leading to maldevelopment and fibrosis. Several strategies reduce the incidence of BPD, including noninvasive ventilation and timely administration of surfactant.^1^

The introduction of surfactant in the late 1980s significantly improved outcomes for premature infants with respiratory distress syndrome (RDS), which is caused by a developmental deficiency of endogenous surfactant in the lungs. The surfactant era coincided with other significant advances for the prevention of BPD, including an extensive use of prenatal steroids and the availability of infant-triggered and high-frequency ventilator modes. Most significantly, advances in noninvasive delivery of positive pressure have altered management strategies in the delivery room and neonatal ICU (NICU). Several large clinical trials have demonstrated a lower relative risk of death or BPD when infants are treated initially with nasal continuous positive airway pressure (CPAP) when compared to prophylactic intubation and surfactant.^2–6^ The American Academy of Pediatrics published a policy statement in 2014 advising an initial noninvasive approach, with subsequent selective surfactant administration.^7^

This recommendation has led to decreased and later use of surfactant. The conflicting need to maximize noninvasive ventilation while also delivering surfactant early for infants with respiratory distress syndrome constitutes a dilemma for clinicians. Some centers have addressed this dilemma by providing surfactant without intubation. However, this method requires additional expertise and is not yet widely available.^8,9^

Increasing time of exposure to high oxygen concentrations via noninvasive ventilation or to positive pressure ventilation before surfactant administration leads to oxygen toxicity, volutrauma, and/or barotrauma to the lung. Therefore, early delivery of surfactant to infants with clinically significant RDS improves outcomes, when compared to those who are treated only after manifesting severe disease.^10–12^ Although evidence identifying the optimal thresholds for intubation and rescue surfactant administration is limited, the establishment of a reasonable unit-based approach can likely improve the quality and uniformity of care for preterm infants.^13,14^ One systematic review indicated that infants receiving surfactant when requiring F_i_O_2_ of 45% had fewer complications than those using higher oxygen thresholds for surfactant administration.^15^

Before this project, initial noninvasive ventilation for premature infants had been enthusiastically adopted in our NICUs. Still, we did not have a standard practice for the indications or timing of rescue surfactant therapy. In baseline data collected from September to December 2016, we found that of 64 infants younger than 32 weeks gestation, 15 (23%) who were admitted on CPAP later required intubation in the NICU. However, 9 (60%) of intubations occurred greater than 2 hours after F_i_O_2_ continuously exceeded 40% (median 161 min), and 10 (67%) of surfactant doses were not administered until greater than 1 hour after intubation. The reasons for late surfactant administration were not usually defined in the electronic medical record (EMR). Still, the most common factors appeared to be the timing of the chest x-ray and the duration of procedures such as line placement. All intubations were considered elective. These delays, which may have contributed to oxygen toxicity and lung injury, constitute an opportunity for the amelioration of a dysfunction in health service delivery that prevents care from reaching its full potential. Although previous investigators have reported on successful quality improvement projects to increase noninvasive ventilation,^13^ to our knowledge, this is the first report targeting delayed surfactant administration in the current environment of ubiquitous noninvasive ventilation.

Our objectives were to implement standard criteria for the diagnosis of RDS in premature infants and decrease the time to intubation and surfactant administration to infants who qualify based on those criteria. The long-term aim is to reduce BPD and other respiratory complications by optimizing the benefits of both noninvasive ventilation and surfactant.

## METHODS

The Institutional Review Board of the Feinstein Institute for Medical Research, Northwell Health, determined that this project was quality improvement and not human subjects research. Therefore, the project did not require review and approval.

### Setting

Cohen Children’s Medical Center (CCMC) NICU is a 57-bed level 4 regional perinatal referral center with 2100 admissions (245 < 1,500 g birth weight, 110 major surgical) annually. North Shore University Hospital (NSUH) NICU is a 52-bed level 3 regional perinatal referral center with 1,100 admissions annually (100 < 1,500 g birth weight).

### Aim

The primary endpoints were to improve the performance of the following measures for newborn infants <1,500 g or younger than 32 weeks gestation:

1. Administer surfactant within 1 hour of intubation for >90% of infants by May 2018.
2. For infants not intubated in the delivery room, intubate within 90 minutes of the start of a continuous run of F_i_O_2_ > 40% or P_a_CO_2_ > 65 (by transcutaneous monitor) by April 2019.

### Measurement

We included infants <1,500 g and/or younger than 32 weeks gestation at birth and obtained F_i_O_2_ from the EMR nursing record. The outcome variable was the time in minutes between qualifying for intubation (start of a continuous run of F_i_O_2_ > 40% or P_a_CO_2_ > 65 for >90 min) and the documented time of intubation. Similarly, we calculated the time in minutes between intubation and surfactant administration from documentation in the EMR.

### Balancing Measures

We quantified the presence of radiographic features of unilateral surfactant administration (asymmetric aeration or atelectasis based on documentation by the attending neonatologist or radiologist), malposition of the endotracheal tube, or pneumothorax on the first chest x-ray (within 24 h) after surfactant administration. These balancing measures assessed the possibility that overly rapid intubation and/or surfactant administration could increase the risk of inappropriate endotracheal tube placement, leading to nonuniform aeration and distribution of surfactant.

### Interventions and Tests of Change

**Fig. 1.**
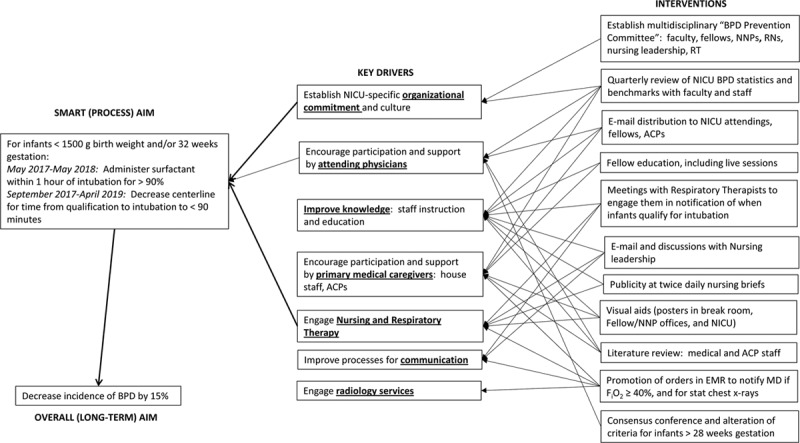
Key driver diagram.

We first established an interdisciplinary “BPD Prevention Team” with representatives from both NICUs and including neonatal attendings, fellows, nurse practitioners, nurse managers, primary nurses, and respiratory therapists. The key drivers were to increase both institutional commitment and awareness of the relevance of the health implications by NICU care providers (Fig. 1).

First, we implemented several plan-do-study-act (PDSA) cycles to decrease the time from intubation to surfactant:

1. PDSA Cycle 1 (May 2017): Email distribution (“high-importance”) to NICU attendings, fellows, and advanced care practitioners (ACPs) outlining the goal to administer surfactant within 1 hour of intubation. NICU leadership frequently uses this method of communication to disseminate clinical guidelines and major announcements and updates in our division.
2. PDSA Cycle 2 (July 2017): New fellow education and email reminders to the medical team, and inclusion of respiratory therapists to aid in notification of when infants qualify for intubation.
3. PDSA Cycle 3 (October 2017): Follow-up email was sent, with distribution expanded to nursing leadership.
4. PDSA Cycle 4 (December 2017–January 2018): The BPD Prevention Team conducted two live sessions to in-service the fellows on the guideline and target for surfactant administration within 1 hour of intubation. Fellows and radiology service developed protocols to communicate the need for an “immediate” chest x-ray. Summary wall hangings were posted in the fellow’s office and the NICU lounge.

In January 2018, the time intervals from intubation to surfactant appeared to be stabilizing. To further improve timely respiratory care for premature infants, we initiated projects to decrease time from qualification to intubation for surfactant administration:

5. PDSA Cycle 5 (January 2018): The BPD Prevention Committee staged a presentation on the importance of early intubation and surfactant administration for those babies who meet criteria at a Neonatal Division Conference targeting neonatal attendings, fellows, and ACPs, followed by distribution of an email summary.
6. PDSA Cycle 6 (March 2018): Announcements were made at morning and evening unit briefs to review criteria for intubation and surfactant administration. We placed posters in the nursing lounge, on-call rooms, and NICU, and conducted ongoing teaching for fellows.
7. PDSA Cycle 7 (April 2018): Literature review and presentation of evidence and our current data at Neonatal Division Conference. Members of the BPD Prevention Team also conducted ongoing teaching during sign-out. They implemented a process for placement of physician orders after the admission of premature infants to remind nurses to notify MD if F_i_O_2_ >40% for 1 hour.
8. PDSA Cycle 8 (July 2018): The BPD Prevention Committee convened a consensus conference with faculty, data reviewed, and intubation criteria adjusted. The F_i_O_2_ threshold was adjusted to 60% for infants older than 28 weeks gestation only. Ongoing education programs for medical staff included small group discussions with new fellows.
9. PDSA Cycle 9 (October 2018): Presentation at the neonatology division process improvement meeting regarding adherence to the BPD bundle and timely intubation, especially at the change of shift, with the initiation of monthly reminders to the medical team.

### Analysis

Comparisons between pre- and postintervention periods for demographic characteristics and respiratory outcomes were made using Wilcoxon rank-sum tests for continuous data and chi-square tests for categorical data. Control charts were plotted and analyzed using QI Macros for Excel (KnowWare International, Denver, Colo.). We identified special cause variations using the Western Electric rules (in this case, 8 consecutive points below the centerline). Also, there were some early points outside the control limits of 3 standard deviations, indicating that the process was unstable before the interventions.

## RESULTS

Table 1 displays infant characteristics and respiratory management outcomes during the 6-month baseline (October 2016–March 2017) and postintervention (October 2018–March 2019) periods. Patient demographics and duration of mechanical ventilation, CPAP, and supplemental oxygen did not differ between the periods.

**Table 1.**
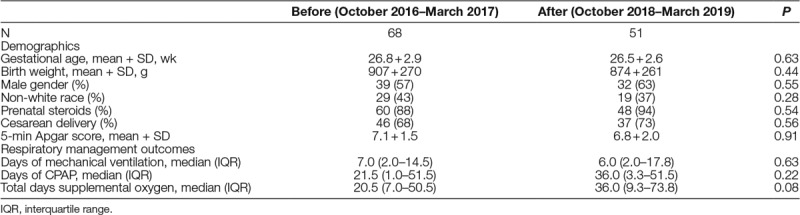
Maternal and Infant Characteristics and Respiratory Management Outcomes at CCMC and NSUH during the 6-month Baseline (October 2016–March 2017) and Postintervention (October 2018–March 2019) Periods

### Time to Surfactant Administration

**Fig. 2.**
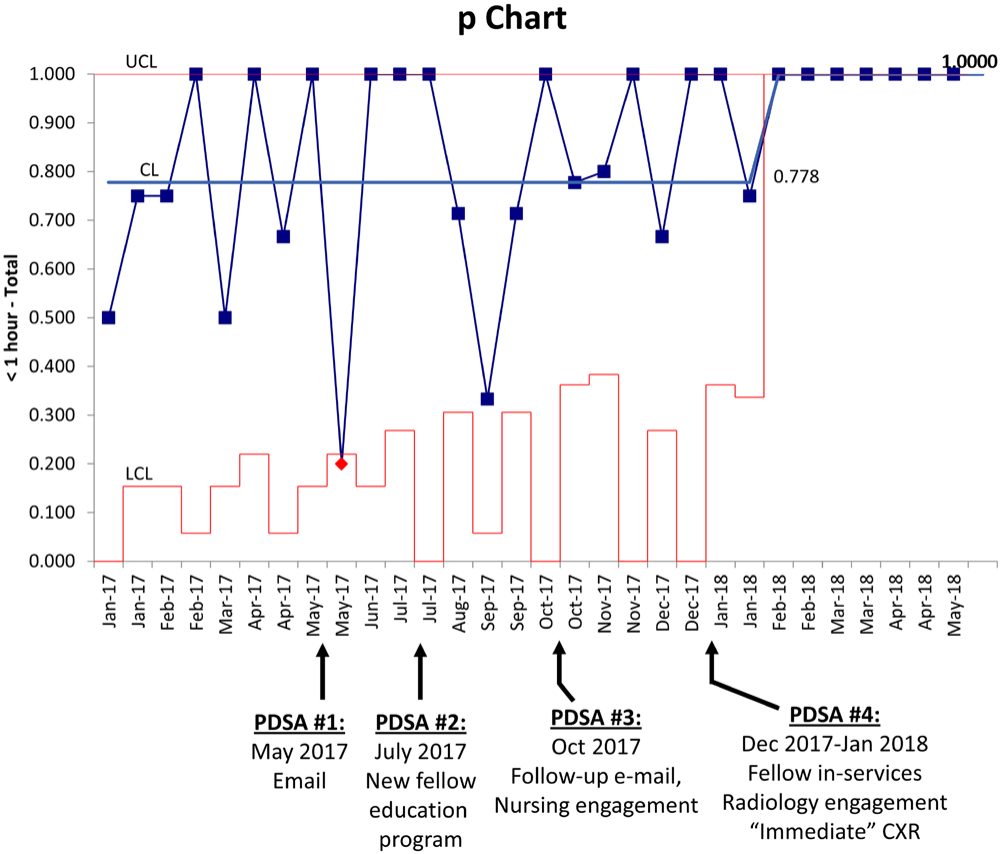
P-chart: proportion of infants receiving surfactant <1 hour from intubation.

Baseline data indicated that only 78% of patients were intubated for <60 minutes before being treated with surfactant (Fig. 2). PDSA #1 (email to NICU attendings, fellows and ACPs), PDSA #2 (new fellow education program), and cycle 3 (another email, inclusion of nursing staff) did not lead to consistent improvement in performance. However, PDSA #4 (a fellow education program) led to rapid improvement, to mean compliance of 100%. No surfactant complications such as pneumothoraces or asymmetric lung expansion secondary to endotracheal tube misplacement (balancing measures) occurred.

### Time to Intubation

**Fig. 3.**
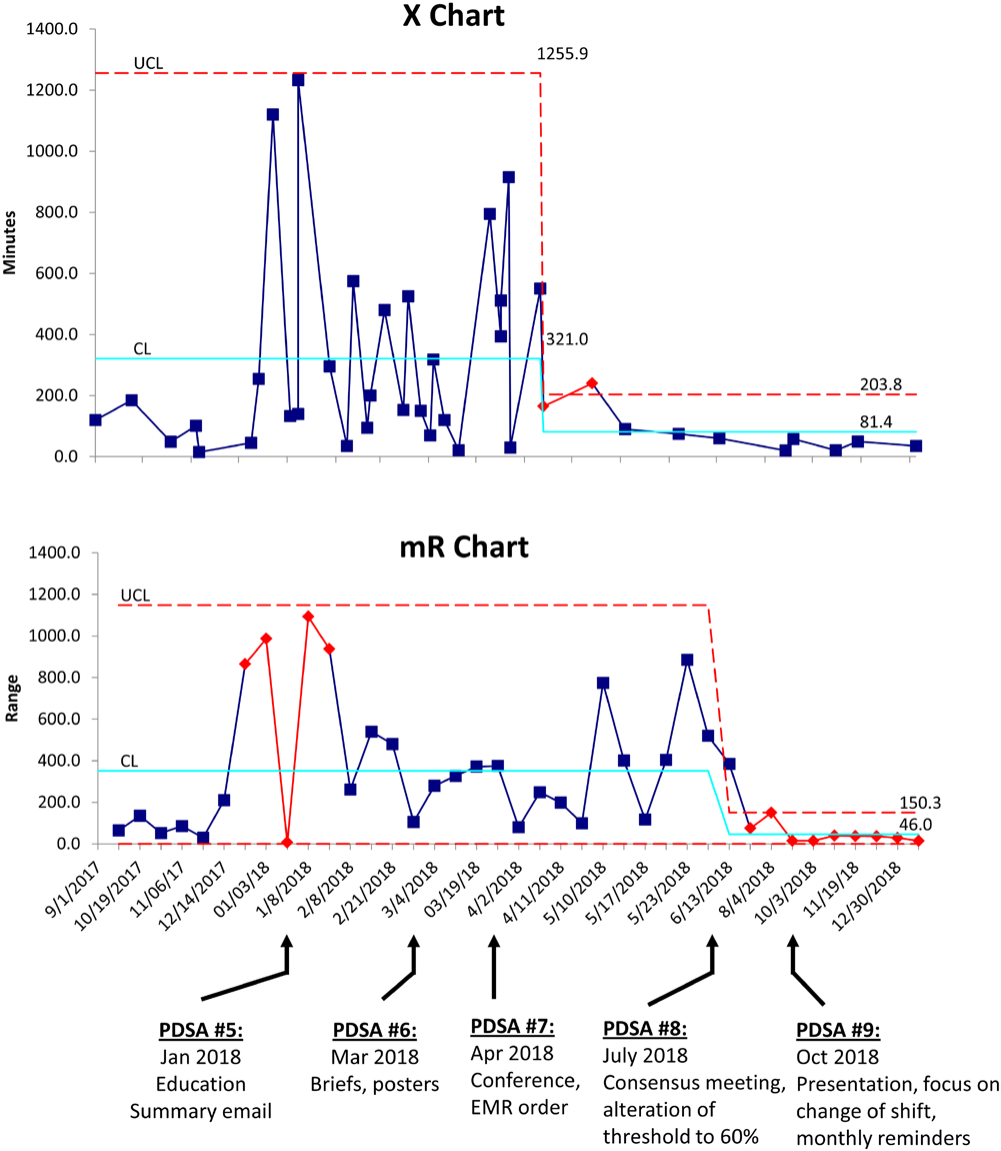
Individuals and moving range (X–mR) chart: Time from target F_i_O_2_ (40%–60%) to intubation (28–32 weeks gestational age). The X–mR chart tracks processes with a subgroup size of one. The individual (X) chart displays individual measurements over time and is used to identify the centerline. The moving range (mR) chart plots the variability between each data point and the next. Both the time to intubation and the stability of the process improved after PDSA #8 for infants 28–32 weeks gestational age.

**Fig. 4.**
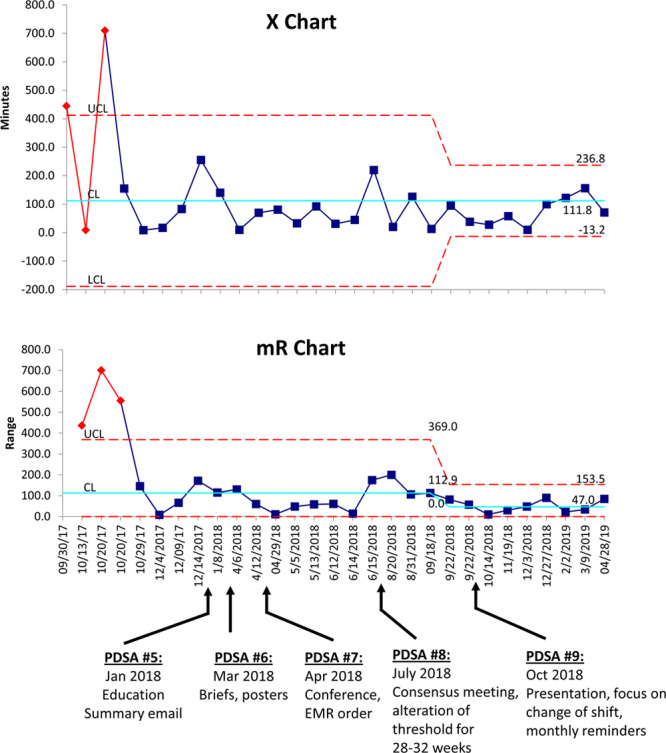
Time from target F_i_O_2_ (40%) to intubation (<28 weeks gestational age). The centerline for time to intubation did not change for infants younger than 28 weeks gestation, but the variability of the process decreased after PDSA #8.

For infants 28–32 weeks gestational age, the baseline mean time from qualification to intubation was 321 minutes, with wide variation between patients. PDSA #5 (general presentations and emails directed at medical and advanced care practitioners) was not effective. PDSA #6 (announcements at unit briefs and placement of visual aids throughout the units) decreased the variability in the time to intubation but did not alter the centerline. PDSA #7 (physician literature review conference and promotion of placing an order in the medical record) led to a decrease in the centerline to 81 minutes, achieving the target. PDSA #8 (a consensus conference of attending physicians and adjustment of the target to F_i_O_2_ = 60%) led to a sustained improvement in compliance. For infants younger than 28 weeks gestation, the baseline mean time to intubation was 112 minutes. This outcome did not decrease in response to the interventions, although the moving range decreased after PDSA #8, indicating less process variability. For all infants in both groups, the mean time to intubation decreased from a centerline of 266–96 minutes (Figs. 3 and 4).

## DISCUSSION

A dedicated BPD Prevention Team and implementation of several cycles of change facilitated timely intubation and administration of surfactant. Time from intubation to administration of surfactant within 1 hour improved to 100%. The median time from threshold to intubation decreased from 321 to 81 minutes for infants 28–32 weeks gestational age, surpassing the target of 90 minutes. It remained 112 minutes for infants younger than 28 weeks, but the mean range decreased.

For surfactant administration, only PDSA #4 was effective (Fig. 2). Delays in obtaining chest x-rays were a frequent barrier to timely administration of surfactant, so we established protocols to communicate the need for an “immediate” chest x-ray from NICU clinicians to radiology technicians. Also, the BPD Prevention Committee intensified instruction for neonatology fellows on the importance of early surfactant and placed wall hangings in their office. The success of these interventions demonstrates the importance of process analysis. After identification of x-ray delays as a key rate determinant, NICU leadership modified processes to prevent them. At the same time, we established that neonatology fellows, rather than attendings or ACPs, administered most surfactant, and intensely targeted education to that group for maximal impact. Educational interventions require an ongoing effort to sustain but are quickly and dramatically effective when appropriately directed.

Similarly, general education, emails, and visual aids to all NICU staff were not effective in reducing time to intubation (Figs. 3 and 4). Process analyses revealed that consultation with attending physicians usually occurred before the decision by fellows or ACPs to intubate infants electively. Therefore, neonatology attendings were the group with the most impact. To reach them and achieve their collaboration, we conducted a division conference, at which medical evidence and our outcome data were reviewed (PDSA #7). A review of the process preceding intubation also revealed that the step most likely to fail was in alerting the physicians about infants approaching qualification. To address this failure, we initiated a new procedure for placement of free-text orders, on admission, for nurses to notify the physicians if F_i_O_2_ > 40% for >1 hour. Subsequently, the median time to intubation for infants 28–32 weeks gestational age decreased from 321 to 81 minutes (Fig. 3).

Despite the success of this approach, the conference made it clear that sustained improvement would require adjustment of the criteria for intubation. We conducted a consensus conference, which resulted in increasing the oxygen threshold to 60% for larger infants (28–32 weeks gestation only), more compatible with the current practice (PDSA #8). This more liberal threshold remained within the bounds of medical evidence for the larger infants, who are at a lower risk for BPD than those younger than 28 weeks gestation. Subsequently, the improvement was sustained, with time to intubation uniformly below the centerline of 81 minutes. This experience demonstrated that a sustained change in fundamental medical practices requires a strong consensus among practitioners and flexibility within the bounds of evidence.

In contrast, the median time to intubation for infants younger than 28 weeks gestation (112 min) did not change in response to any of the projects, and we did not achieve the target of 90 minutes (Fig. 4). Delays in intubation in this particularly vulnerable group may be related to other concurrent needs, such as placement of central lines. This possibility will be addressed in ongoing projects. However, it is essential to note that the process became more stable, as reflected in decreased mean range for time to intubation after PDSA #8 (consensus conference and alteration of the intubation threshold for larger infants). This decrease may be due to a “halo” effect related to the consensus conference. The accommodation of alternative management strategies for the larger infants may have led to increased “buy-in” by physicians, potentially improving care for the smaller infants as well.

We recognize that several of the improvement interventions described are based on education and consensus, rather than a process change. As such, they have poor reliability. Nevertheless, their rapid, successful impact was both surprising and encouraging. We repeated several of the interventions, such as emails and announcements at briefs, repeatedly during the intervals between PDSA cycles to increase their efficacy. In addition to ongoing education, the committee implemented several targeted interventions to increase the likelihood of sustained improvement. For example, PDSA #9 included a focus on time around hand-off at the change of shift, when delays were most prevalent. We expect that these approaches can be generalized to other populations because the extremely high patient census and turnover in NICUs make them ideal laboratories for evaluating quality improvement strategies in high-complexity and/or high-intensity inpatient environments.

From 2017 to 2018, the incidence of chronic lung disease (as defined by Vermont Oxford network reporting) decreased from 28.6% to 22.3% at CCMC, and from 25.6% to 21.8% at NSUH. The etiology of BPD is multifactorial, and any effective unit improvement plan should address multiple risk factors. Although it is not possible to assign causation to these interventions, they may have contributed.

Although the benefits of surfactant for premature infants with respiratory distress syndrome are well established, its use has decreased and become more remote from the initial indication in the current era of noninvasive ventilation. Our findings suggest that the implementation of standard criteria for intubation and surfactant administration is feasible and can facilitate timely therapy, potentially decreasing the adverse outcomes and increased utilization of healthcare resources associated with bronchopulmonary dysplasia.

## DISCLOSURE

The authors have no financial interest to declare in relation to the content of this article.
